# Efficient ethanol production from dried oil palm trunk treated by hydrothermolysis and subsequent enzymatic hydrolysis

**DOI:** 10.1186/s13068-015-0263-6

**Published:** 2015-06-11

**Authors:** In-Yong Eom, Ju-Hyun Yu, Chan-Duck Jung, Kyung-Sik Hong

**Affiliations:** Center for Bio-based Chemistry, Green Chemistry & Engineering Division, Korea Research Institute of Chemical Technology, 141 Gajeong-ro, Yuseong-gu, Daejeon, 305-600 Republic of Korea

**Keywords:** Oil palm trunk, Starch, Hydrothermal treatment, Enzymatic hydrolysis, Ethanol fermentation

## Abstract

**Background:**

Oil palm trunk (OPT) is a valuable bioresource for the biorefinery industry producing biofuels and biochemicals. It has the distinct feature of containing a large amount of starch, which, unlike cellulose, can be easily solubilized by water when heated and hydrolyzed to glucose by amylolytic enzymes without pretreatment for breaking down the biomass recalcitrance. Therefore, it is suggested as beneficial to extract most of the starch from OPT through autoclaving and subsequent amylolytic hydrolysis prior to pretreatment. However, this treatment requires high capital and operational costs, and there could be a high probability of microbial contamination during starch processing. In terms of biochemical conversion of OPT, this study aimed to develop a simple and efficient ethanol conversion process without any chemical use such as acids and bases or detoxification.

**Results:**

For comparison with the proposed efficient ethanol conversion process, OPT was subjected to hydrothermal treatment at 180 °C for 30 min. After enzymatic hydrolysis of PWS, 43.5 g of glucose per 100 g dry biomass was obtained, which corresponds to 81.3 % of the theoretical glucose yield. Through subsequent alcohol fermentation, 81.4 % ethanol yield of the theoretical ethanol yield was achieved. To conduct the proposed new process, starch in OPT was converted to ethanol through enzymatic hydrolysis and subsequent fermentation prior to hydrothermal treatment, and the resulting slurry was subjected to identical processes that were applied to control. Consequently, a high-glucose yield of 96.3 % was achieved, and the resulting ethanol yield was 93.5 %.

**Conclusions:**

The proposed new process was a simple method for minimizing the loss of starch during biochemical conversion and maximizing ethanol production as well as fermentable sugars from OPT. In addition, this methodology offers the advantage of reducing operational and capital costs due to minimizing the process for ethanol production by excluding expensive processes related to detoxification prior to enzymatic hydrolysis and fermentation such as washing/conditioning and solid–liquid separation of pretreated slurry. The potential future use of xylose-digestible microorganisms could further increase the ethanol yield from the proposed process, thereby increasing its effectiveness for the conversion of OPT into biofuels and biochemicals.

## Background

Recently, lignocellulosic biomass has become recognized for its potential as a renewable natural resource for producing biofuels and biochemicals due to rising interest in exploring alternatives to fossil fuels and reducing global warming. Among the promising lignocellulosic biomass resources, oil palm trunk (OPT) has been spotlighted as a valuable energy resource due to the increased production of palm oil. The palm oil production in from 2012 to 2013 was 28.5 million tons in Indonesia and 18.5 million tons in Malaysia, which amounts to approximately 88 % of the worldwide palm oil production. Moreover, palm oil production is expected to continue growing as the biodiesel industry expands due to the rising demand for palm oil as the raw material for biodiesel [1, 2]. To harvest fresh palm with its high-oil productivity, the oil palm tree is recommended to be replanted at intervals of 20–25 years. Consequently, a large amount of OPT is inevitably generated as a by-product in the process of rebuilding plantation sites. In Malaysia, for example, oil palm trees cultivated in 500,000 ha are annually cut for replanting. In such oil palm plantations, approximately 70 million palm trees are generated annually, affording more than 15 million tons OPT [3]. Some parts of OPT such as its hard outer layer were often used for plywood manufacturing, but most of it tends to be discarded or burnt [4]. Recently, research has focused on the use of OPT as a main feedstock for conversion into biofuels such as ethanol and biochemicals in terms of biorefinery concept [4–8].

OPT, unlike general woody biomass, has distinct compositional characteristics. The flesh of OPT contains sap, which accounts for up to 80 % of the felled OPT, depending on its parts. The sap characteristically consists of diverse free sugars, among which glucose is dominant, followed in order by sucrose and fructose, and also small amounts of other sugars [7]. Amino acids, organic acids, minerals, and vitamins are also present in the sap. Hence, some studies reported that the sap recovered by squeezing OPT could be directly fermented into either biofuels or biochemicals such as ethanol and lactic acid, respectively [4–7]. The OPT bagasse, after squeezing the sap, remains approximately 30 % based on wet felled trunk, and it also can be used as feedstock in biorefining. It still contains a considerable amount of starch and cellulose accounting for around 50 % on a dry weight basis. Interestingly, it can be fractionated into parenchyma (PA) enriched with starch and vascular bundle (VB) of acicular fibers during its pulverizing [8].

Besides starch, dried OPT also consists of cell wall components such as cellulose, hemicelluloses, and lignin. However, the structural components of OPT, unlike those of palm trunk sap, cannot be easily converted by biochemical processes due to biomass recalcitrance. Therefore, disrupting the biomass recalcitrance is a prerequisite for converting OPT into the desired chemicals. Hence, a number of pretreatment technologies capable of breaking down the biomass recalcitrance have been developed, which makes cellulose more accessible to cellulolytic enzymes. Among the various pretreatment methods, hydrothermal treatment, also called liquid hot water or autohydrolysis, requires lower capital and operational expenses due to the use of only water as the reaction medium without any chemicals such as acids or bases. Moreover, hydrothermal treatment can reduce the unit process related to the chemical recovery and conditioning for subsequent enzymatic hydrolysis thanks to the rare generation of inhibitory products such as furfural, 5-hydroxymethyl-2-furaldehyde (HMF), and phenolics during the process [9]. To date, a number of previous studies showed that hydrothermal treatment has a favorable effect on increasing the enzymatic digestibility of a variety of herbaceous biomasses, including switchgrass, sunflower stalks, and wheat straw [10–14]. Moreover, Inbicon A/S has developed an integrated biomass utilization system for a large-scale biorefinery process to produce ethanol from lignocellulosic biomass by using an efficient continuous hydrothermal system [14].

Due to the use of only water as the reaction medium, without the need to add any acid or base catalysts, particularly, hydrothermal treatment is suitable for pretreating some starch-rich lignocellulosic biomasses such as OPT since most of the starch can be solubilized under less severe conditions such as autoclaving at 121 °C for 15 min while minimizing its further degradation [7]. In an acidic medium containing sulfuric acid ranging from 0.15 to 0.7 % (*w*/*w*), on the contrary, it has been reported that further degradation of starch to HMF occurs above 150 °C within a few minutes [15]. In such a case, when OPT is subjected to acid pretreatment, starch loss could be inevitable.

For effective conversion of starch and cellulose in the dried OPT into ethanol, a newly designed and simple method is therefore proposed to minimize starch loss during biochemical conversion and to maximize the production of ethanol and fermentable sugars from OPT. This study also evaluated how much more ethanol production from OPT was increased through the newly designed process than that via conventional process in which OPT was applied to hydrothermal treatment, followed by enzymatic hydrolysis and ethanol fermentation of pretreated OPT slurry.

## Materials and methods

### Raw material

The dried OPT chips (*Dami*) were kindly supplied by the Korindo Group (Jakarta, Indonesia). The feedstock was ground using a knife mill equipped with a 20-mesh aperture screen, and the powder containing 5.4 % moisture was stored at −20 °C in a refrigerator in sealed plastic bags until use. Sulfuric acid (72 % *w*/*w*, Sigma-Aldrich (St. Louis, MO, USA)) as reagent for compositional analysis was purchased commercially. Cellic® CTec2 as a cellulase complex and Cellic® HTec2 as xylanase were purchased from Novozymes Korea (Seoul, Korea), and Novozyme-188 as a supplement to enhance cellobiase activity was purchased from Sigma-Aldrich (St. Louis, MO, USA). Glucoamylase and α-amylase were purchased from Sigma-Aldrich (St. Louis, MO, USA) for enzymatic hydrolysis of starch. Enzyme activities used in this study are described in Table 1.

**Table 1:** Enzyme activities used in this study

Enzymes	Enzyme activity		
	Cellulase (FPU/g)^a^	Cellobiase (CBU/g)^a^	Amylase (U/g)^b^
Cellic® CTec2	106 ± 7	3,228 ± 250	31 ± 1
Novozyme-188	n.d.	554 ± 28	1,482 ± 33
α-Amylase	n.d.	3.9 ± 0.1	18,769 ± 523
Glucoamylase	n.d.	n.d.	3,475 ± 124

*n.d.* not detected

^a^Activities were determined by the filter paper and cellobiase assays [22]

^b^Activity was represented by the total amylolytic activity [23]

### Compositional analysis

The starch content was determined after converting starch to glucose via enzymatic hydrolysis by amylolytic enzyme mixtures consisting of glucoamylase and α-amylase with a volumetric ratio of 9 to 1. To solubilize starch, 120 g of dry OPT was loaded into a 3-L-scale batch-type fermenter (BioTron, Seoul, Korea) and then filled with deionized water to a total weight of 1500 g. This soaked slurry was autoclaved at 121 °C for 20 min and cooled down at 50 °C, after which amylolytic enzymes corresponding to 5 μL per 1 g dry biomass were added. Enzymatic hydrolysis of starch was conducted at 50 °C for 24 h with stirring. The liberated glucose was quantified using high-performance liquid chromatography (HPLC) equipped with an autosampler (Waters 2707 (Waters, Milford, MA, USA)), a refractive index detector (Waters 2414 (Waters, Milford, MA, USA)), and a binary HPLC pump (Waters 1525 (Waters, Milford, MA, USA)). An Aminex HPX-87H column (BIO-Rad, Hercules, CA, USA) operating at 50 °C was used, and the mobile phase was 5 mM H_2_SO_4_, at a flow rate of 0.6 mL/min. The remaining solid was washed with deionized water, and then centrifuged several times until glucose was not detected without loss of the solid, and then the solid was freeze-dried. The dried solid was pulverized, and its moisture content was determined by oven drying at 105 °C for 48 h. The composition of structural components of starch-free OPT was determined according to National Renewable Energy Laboratory (NREL) standard procedures [16, 17]. The monosaccharides were determined using HPLC. The acid-soluble lignin was also determined by DU 800 UV/Vis spectrophotometer (Beckman Coulter Inc., Fullerton, CA, USA) at 320 nm. The acid-insoluble lignin on the crucibles was gravimetrically determined, based on the remaining insoluble residue, by subtracting the ash content from it. All compositional analyses were run in triplicate and reported as average and standard deviation.

### Two types of ethanol conversion process

**Fig. 1:**
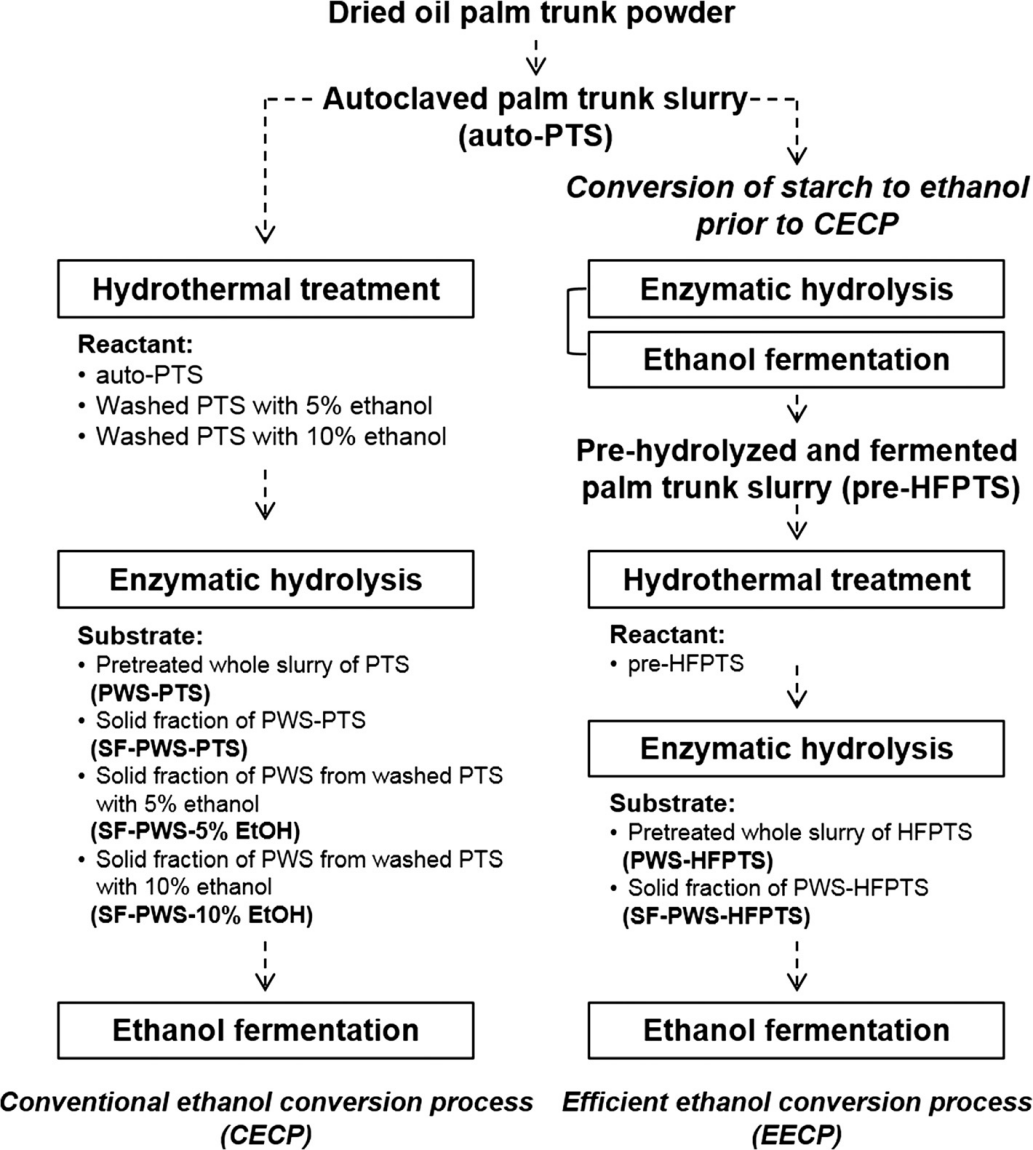
Flow diagrams of the two types ethanol conversion process

Ethanol was produced from OPT by two types ethanol conversion processes, which were evaluated in terms of the yields of fermentable sugar and ethanol. As shown in Fig. 1, the conventional ethanol conversion process (CECP), which includes soaking of OPT, is termed autoclaved palm trunk slurry (auto-PTS), hydrothermal treatment of auto-PTS, and subsequent fermentation of whole slurry. The newly designed and simple method proposed herein, termed the efficient ethanol conversion process (EECP), is suitable for effective biochemical conversion of starch-rich lignocellulosic biomass such as OPT to ethanol. To minimize starch loss in the OPT, pre-hydrolysis and fermentation of starch was carried out prior to the CECP. Particularly, both EECP and CECP had the distinctive feature that enzymatic hydrolysis and fermentation of pretreated whole slurry (PWS) were carried out without solid–liquid separation, washing, and detoxification after hydrothermal treatment of OPT.

### Conversion of starch to ethanol

Starch in OPT was preferentially converted to ethanol through enzymatic hydrolysis and subsequent ethanol fermentation prior to hydrothermal treatment according to the following detailed method. The OPT equivalent to 120 g of dry biomass loaded in a 3-L-scale batch-type fermenter (BioTron, Seoul, Korea) was filled with deionized water to a total weight of 800 g and then it was autoclaved at 121 °C for 20 min to solubilize starch from the OPT. After autoclaving, amylolytic enzyme mixtures amounting to 5 μL per 1 g dry biomass were added to the fermenter. These enzyme mixtures were prepared by glucoamylase (4170 U/mL) and α-amylase (22,523 U/mL) with a volumetric ratio of 9 to 1. The slurry was filled with deionized water to a concentration of 10 % (*w*/*w*). Enzymatic hydrolysis of starch was carried out at 50 °C with a stirring speed of 200 rpm for 24 h. Seed culture of *Saccharomyces cerevisiae* (24858 ATCC) was carried out in YPD medium consisting of 5 g/L yeast extract, 10 g/L peptone, and 25 g/L glucose at 30 °C in a rotary shaker at 200 rpm. After 24-h enzymatic hydrolysis, 5 % (*v*/*v*) yeast inoculum was added into enzymatic hydrolysates. No additional nutrients were added to the hydrolysates except on seed culture medium. The fermentation was carried out at 30 °C with a stirring speed of 200 rpm for 24 h. The pH was kept at 5 by adding 15 % ammonia solution. After completion of starch hydrolysis and ethanol fermentation, each aliquot of 1 mL was scooped from well-mixed slurries and separated by centrifugation. Each of the glucose and ethanol in the supernatants was determined using HPLC. The pre-hydrolyzed and fermented PTS (pre-HFPTS) was sequentially used as a material for EECP.

### Hydrothermal treatment and subsequent enzymatic hydrolysis

To determine pretreatment temperatures suitable for maximizing hemicellulosic sugar yield from OPT through enzymatic hydrolysis of whole slurry, hydrothermal treatment was performed using a bomb-type mini-reactor. The detailed procedures on the hydrothermal treatment and enzymatic hydrolysis have been previously described [12]. OPT powder equivalent to 1 g dry biomass was placed on a reactor vessel, to which deionized water (20 mL) was added, followed by keeping it overnight. Hydrothermal treatment was carried out at temperatures ranging from 160 to 200 °C at intervals of 10 °C for 30 min, respectively. The enzymatic hydrolysis procedure is briefly described as follows. The pretreated whole slurries were completely transferred to Erlenmeyer flasks (125 mL). Enzyme mixtures of Cellic® CTec2 and Cellic® HTec2 supplement with or without Novozyme-188 (volumetric ratio of 9:1:0.5) were loaded on pretreated slurries with the ratio of 0.1 mL enzyme mixtures per g dry biomass. To control pH and prevent microbial contamination, 0.05 M sodium citrate buffer (pH 5) and 330 mg/L sodium azide were used, respectively. The Erlenmeyer flasks were filled with deionized water to a total working weight of 40 g and placed in a shaking incubator set to 50 ± 1 °C with a rotating speed of 200 rpm for 72 h. After 72 h, 1 mL aliquots were scooped from well-mixed hydrolyzed slurry and separated by centrifugation. Monomeric sugars in the supernatant were determined by HPLC.

### Effect of solid–liquid separation of pretreated whole slurry (PWS) on enzymatic digestibility

To investigate the effect of solid–liquid separation of PWS on enzymatic digestibility, two kinds of PWS were prepared as follows: auto-PTS and pre-HFPTS, which were subjected to hydrothermal treatment at 180 °C for 30 min using a mini-reactor, respectively. The PWSs from the auto-PTS and pre-HFPTS were referred to as PWS-PTS and PWS-HFPTS, respectively. These PWSs were completely transferred to a 50-mL falcon tube and centrifuged at 3727 × *g* for an hour with a swing-type centrifuge. The recovered liquid fractions (LFs) from the PWS-PTS and PWS-HFPTS were referred to as LF-PWS-PTS and LF-PWS-HFPTS, respectively. The 1-mL aliquots of each supernatant were analyzed by HPLC to determine monomeric sugars. To determine liberated monomeric sugars via 4 % acid hydrolysis, in addition, 5-mL aliquots of LF-PWS-PTS and LF-PWS-HFPTS were transferred to pressure vessels (25.4-mm O.D. × 114-mm L with a capacity of 15 mL, Chemglass, VWR International, IL, USA) with the addition of 8 % (*w*/*w*) sulfuric acid (5 mL) and autoclaved at 121 °C for an hour. The acid-hydrolyzed LFs (AHLFs) from the PWS-PTS and from the PWS-HFPTS were referred to as AHLF-PWS-PTS and AHLF-PWS-HFPTS, respectively.

To recover the solid fractions (SFs), remaining PWSs of PWS-PTS and PWS-HFPTS after decanting LFs were filtered with Whatman GF/D glass fiber filter in a 250-mL Buchner funnel with vacuum and washed with 100 mL of deionized water to remove any remaining liquid and pre-fermented ethanol. The recovered SFs from the PWS-PTS and PWS-HFPTS were referred to as SF-PWS-PTS and SF-PWS-HFPTS, respectively. The retained SFs and finely scissored filter were completely transferred to Erlenmeyer flasks (125 mL).

Meanwhile, to investigate the effect of ethanol concentration converted from starch on the hydrothermal treatment performance and resulting glucose yield through enzymatic hydrolysis of cellulose, the starch-free OPT obtained from auto-PTS washed with deionized water was added to a mini-reactor and then filled with 20 mL of 5 and 10 % (*w*/*v*) ethanol solution, which was subjected to pretreatment at 180 °C for 30 min, respectively. These PWSs from the starch-free OPT with 5 and 10 % (*v*/*v*) ethanol were separated into LFs and SFs, respectively. The recovered SFs were termed SF-PWS-5 % EtOH and SF-PWS-10 % EtOH, respectively, and were subjected to enzymatic hydrolysis.

### Evaluation of up-scaled ethanol conversion processes through CECP and EECP

Ethanol was produced from OPT by two kinds of laboratory-scale ethanol conversion processes. OPT underwent hydrothermal treatment using a total volume of 2-L-scale stirred Parr reactor (Parr Instruments, Moline, IL, USA). In CECP, the 120 g of OPT (dry biomass basis) loaded in a 1-L media bottle (Schott Duran, Mainz, Germany) was filled with deionized water to a concentration of 15 % (*w*/*v*) and then autoclaved at 121 °C for 20 min. The auto-PTS was transferred to a reactor vessel and then adjusted with deionized water to a total weight of 1500 g corresponding to a concentration of 8 % (*w*/*v*). In EECP, the pre-HFPTS was completely transferred into a reactor vessel as mentioned above. The auto-PTS and pre-HFPTS were subjected to hydrothermal treatment at 180 °C for 30 min. After pretreatment, each slurry was completely transferred into a 3-L-scale batch-type fermenter to which an enzyme mixture of Cellic® CTec2, Cellic® HTec2, and Novozyme-188 (volumetric ratio of 9:1:0.5) corresponding to 0.1 mL per g dry biomass was loaded. Enzymatic hydrolysis was carried out at 50 °C with a stirring speed of 200 rpm for 72 h. The pH was kept at 5 by adding 15 % aqueous ammonia throughout the 72-h hydrolysis. The subsequent ethanol fermentation was performed in the same aforementioned way.

## Results and discussion

### Compositional characteristic of the dried oil palm trunk (OPT)

The OPT compositions are presented in Table 2. Hot-water extractives (HWE) accounting for 34 % of the dried OPT could be extracted with autoclaving at 121 °C for 20 min, but no monomeric sugars were present. When subjected to acid hydrolysis, HWE gave 26.9 g glucose per 100 g dry biomass. It was liberated from the starch that constitutes 80 % of the HWE. Other components constituting the cell wall structure were cellulose (26.6 g as glucose), hemicellulose (17.8 g as monomeric sugars), and lignin (18.9 g). The available glucose from the OPT was 53.5 g, but half of this (26.6 g) could be obtained from the cellulose through pretreatment and subsequent enzymatic hydrolysis. The 26.9 g remainder could be produced through a simple method such as starch hydrolysis without any thermochemical pretreatment. If OPT is applied to pretreatment with high severity, the starch could be further degraded to inhibitory products against microbial fermentation and also acts as a feedback inhibitor to hinder cellulolytic enzymes. To maximize ethanol yield from OPT, therefore, it is crucial to pretreat OPT under a less severe condition to minimize starch degradation.

**Table 2:** Composition of dried oil palm trunk (OPT)

Component	Composition (g)^a^
Cellulose as glucose	26.6 ± 0.2
Hemicellulosic sugars	17.8 ± 0.1
Xylose + galactose + mannose (XGM)	15.4 ± 0.1
Arabinose	2.4 ± 0.1
Acetyl group	3.4 ± 0.0
Acid-insoluble lignin	18.7 ± 0.2
Acid-soluble lignin	0.2 ± 0.0
Hot-water extractives^b^	34.0 ± 0.0
Starch as glucose	26.9 ± 0.1
Ash	5.2 ± 0.1

^a^Average result of triplicate run on 100 g dry biomass basis

^b^Hot-water extractives containing the starch

### Hydrothermal treatment at different temperatures and subsequent enzymatic hydrolysis of whole slurry

**Fig. 2:**
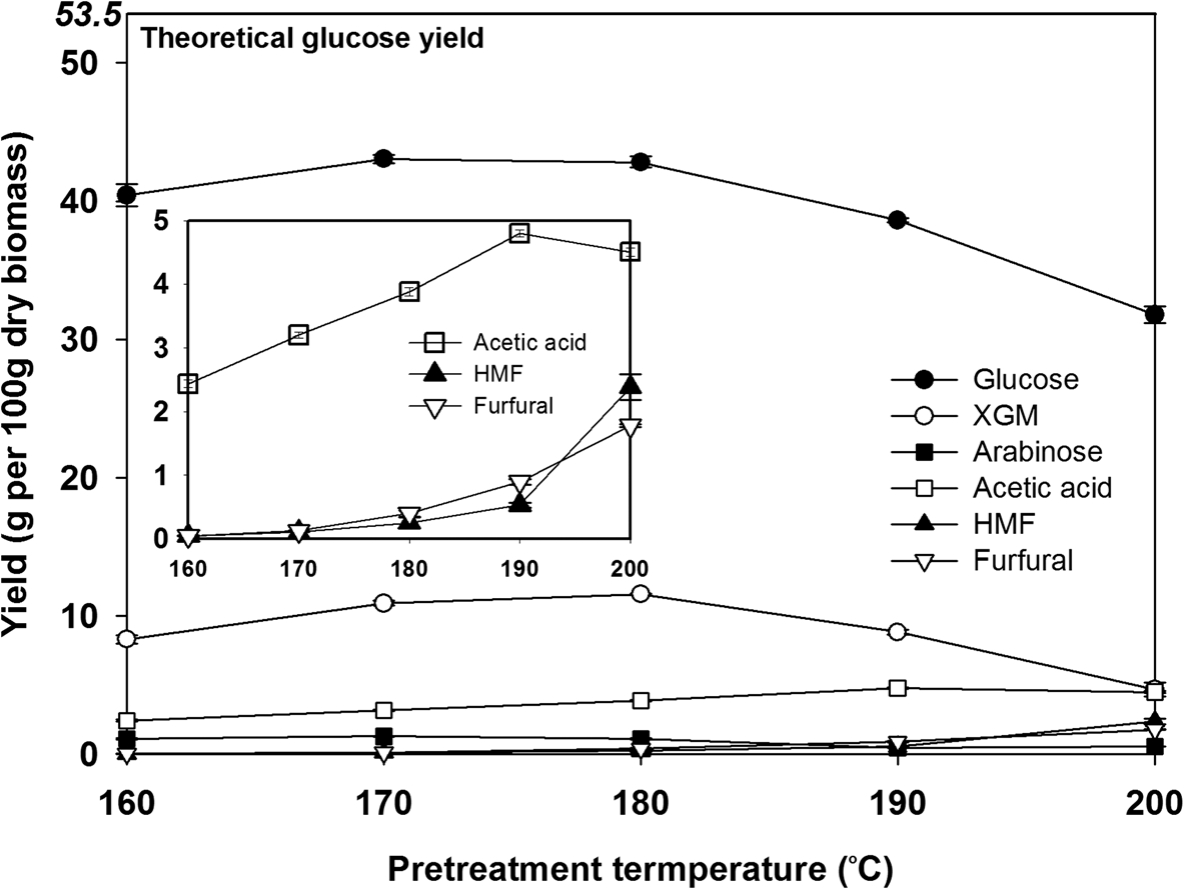
Product yields from enzymatic hydrolysis of whole slurry hydrothermally treated at temperatures ranging from 160 to 200 °C

Figure 2 shows the product yields from enzymatic hydrolysis of PWSs. The production of hemicellulosic sugars, including the sum of xylose, galactose, and mannose expressed as XGM, was maximized from the PWS-PTS at 180 °C and reached 11.6 g per 100 g dry OPT, which was 75.3 % of the theoretical XGM yield (TXY). However, the XGM yield decreased to 4.5 g (corresponding to 29.2 % of TXY) with increasing temperatures above 180 °C. In accordance with the XGM yield, the glucose yield was reached to 43.5 g from PWS-PTS at around 180 °C, which corresponds to just 81.3 % of the theoretical glucose yield (TGY) of 53.5 g. As the OPT was hydrothermally treated at higher temperatures above 180 °C, the glucose yield significantly decreased to 31.8 g corresponding to 59.4 % of TGY. As shown in Fig. 2, the yield lines of an acetic acid, HMF, and furfural-inhibiting enzymes and microorganisms are also shown. With increasing temperatures, particularly, both furfural and HMF, originating from further decomposition of carbohydrates, were exponentially generated. These results differ from those of previous studies on the hydrothermal treatment of lignocellulosic biomass such as wheat straw and sunflower stalks [10, 12, 18]. According to previous studies, the yield of hemicellulosic sugars could be maximized at lower temperatures ranging 170–190 °C, at which their further degradation rarely occurred, whereas high enzymatic digestibility of cellulose could be achieved when lignocellulosic biomass was hydrothermally treated at relatively higher temperatures in the range of 190–220 °C, at which temperature the severe condition inevitably leads to the generation of further degradation products such as furfural and acetic acid from hemicelluloses [12]. As shown in Fig. 2, however, when OPT was hydrothermally treated at temperatures elevated from 180 to 200 °C, the glucose yield gradually decreased from 42.8 to 31.8 g per 100 g dry biomass, but HMF, which is known as a degradation product from glucose, significantly increased around ten times, from 0.25 per 100 g dry biomass at 180 °C to 2.38 g at 200 °C. When LFs after hydrothermal treatment at from 180 to 200 °C were characterized, the generated pH was decreased from 4.1 to pH 3.4, and the amount of liberated glucose via 4 % acid hydrolysis of the LF was decreased from 25.2 to 17.3 g (data not shown). Considering these results, with increasing pretreatment severity, some of the solubilized starch are further degraded to HMF or organic acids, so total glucose yield from the OPT decreased.

To produce ethanol at a laboratory-scale, therefore, OPT was subjected to hydrothermal treatment at 180 °C for 30 min, which yielded the maximum hemicellulosic sugars with a low concentration of furfural and HMF. However, the hydrothermal treatment under mild conditions resulted in an unfavorable glucose yield of around 80 % of TGY, which was overcome in the present study by designing a new upstream process for maximizing starch utilization prior to hydrothermal treatment. The relating results are discussed in detail below.

### Comparative study of the two types of ethanol conversion process

A comparative study on CECP and EECP depicted in Fig. 1 was carried out to evaluate in terms of both fermentable sugar and ethanol yield from OPT. CECP consists of the hydrothermal treatment, enzymatic hydrolysis, and ethanol fermentation of the whole slurry. In EECP, on the other hand, enzymatic hydrolysis and ethanol fermentation of starch in OPT prior to the hydrothermal treatment were added to the CECP. In both processes, fermentable sugars and ethanol were produced from OPT by using only water as a reaction medium and without any solid–liquid separation.

### Effect of solid–liquid separation of PWS-PTS and PWS-HFPTS on glucose yield

**Fig. 3:**
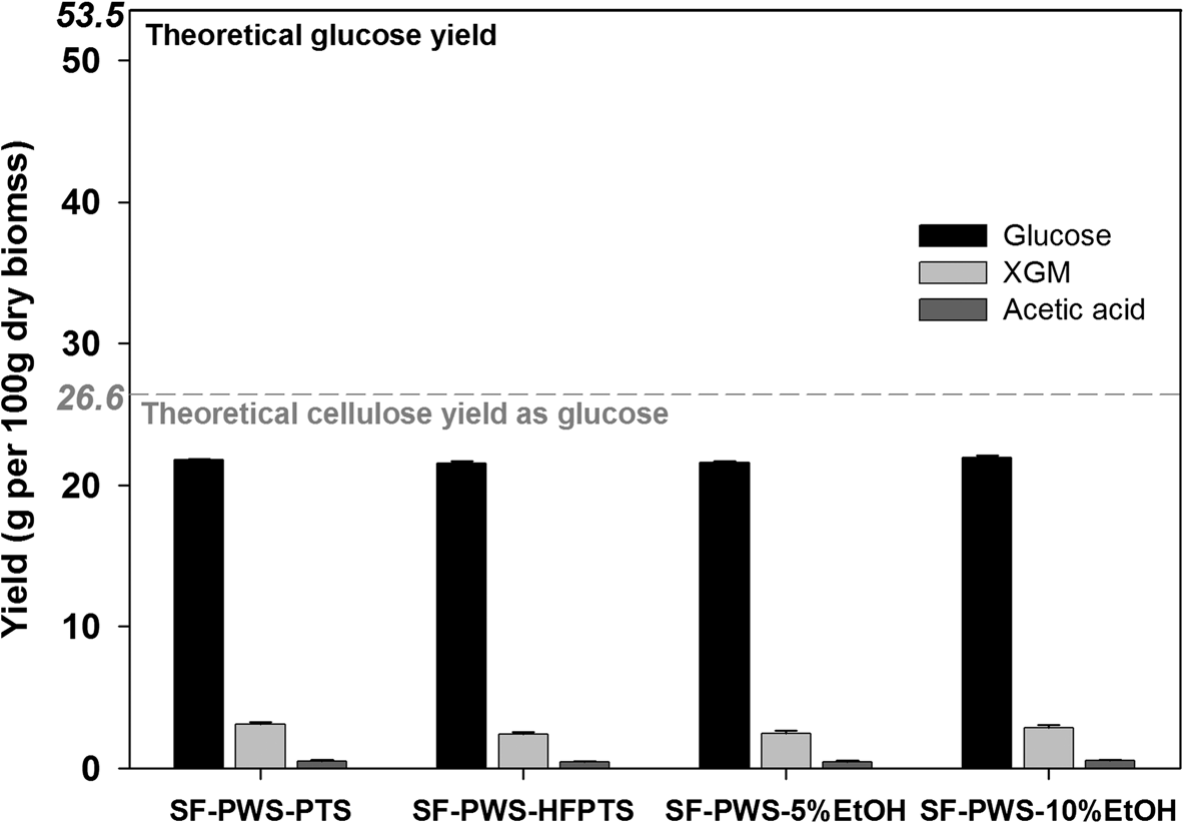
Product yields from solid fractions (SFs) of pretreated whole slurry (PWS) from autoclaved palm trunk slurry (auto-PTS), pre-hydrolyzed and fermented palm trunk slurry (pre-HFPTS), and starch-free oil palm trunk (starch-free OPT) pretreated with 5 and 10 % ethanol solutions through enzymatic hydrolysis

To investigate the effect of solid–liquid separation of PWS on its cellulose digestibility, SFs from auto-PTS and pre-HFPTS were used as a substrate for enzymatic hydrolysis. To investigate whether pretreatment performance was affected depending on ethanol concentration formed from starch fermentation, moreover, enzymatic hydrolysis of SFs from starch-free OPT treated in the presence of ethanol was conducted. These results can be shown in Fig. 3.

No significant change was observed in glucose yields from either SF-PWS-5 % EtOH or SF-PWS-10 % EtOH compared with those from SF-PWS-PTS and SF-PWS-HFPTS, which implies that the conversion of starch to ethanol prior to hydrothermal treatment had no adverse effect on pretreatment performance. However, when SF-PWS-PTS and SF-PWS-HFPTS were subjected to enzymatic hydrolysis, both resulting glucose yields were only around 21.5 g accounting for 40.2 % of TGY, which indicated that conversion of starch to ethanol prior to hydrothermal treatment had no particular effect on cellulose digestibility. In the case using sunflower stalks, contrary to OPT, the glucose yield after solid/liquid separation of pretreated slurry via enzymatic hydrolysis was more than 10 % than that from the pretreated slurry [12]. The increase in glucose yield after solid/liquid separation was due to the removal of liquid fraction of the pretreated slurry. Generally, enzymatic hydrolysis and microbial fermentation of whole slurry tend to be inhibited by further degraded products from hemicellulose and lignin, which are solubilized in the liquid during biomass pretreatment [19]. In addition, the liquid of PWS contains a large amount of xylooligosaccharides due to partial hydrolysis and solubilization of hemicelluloses, which are well known as strong inhibitors for cellulolytic enzymes [19, 20]. However, this study has observed that although each SF from solid–liquid separation of PWS-PTS and PWS-HFPTS was subjected to enzymatic hydrolysis, their glucose yields were around 21.5 g, which was significantly lower than that from PWS (42.8 g), as shown in Fig. 3. In accordance with this result, a previous study has also shown that when enzymatic hydrolysis was carried out using OPT treated with aqueous ammonia and then washed with water, high enzymatic digestibility of more than 90 % was achieved from pretreated solid. However, glucose of less than 30 g could be obtained from 100 g dry raw biomass containing more than 55 g available glucose [21]. Considering the above result, the lower glucose yield from SFs compared to that from PWS was attributed to the removal of LF by solid–liquid separation of PWS. This indicates that a large portion of the starch was solubilized in LF during hydrothermal treatment; thus, the 21.5 g of glucose could be obtained from the remaining cellulose in washed SF via its enzymatic hydrolysis, which is equivalent to 80.8 % of theoretical cellulose yield as glucose. Therefore, enzymatic hydrolysis of PWS could be essential for maximizing fermentable sugar when starch containing biomass such as OPT is used in a biorefining process.

**Fig. 4:**
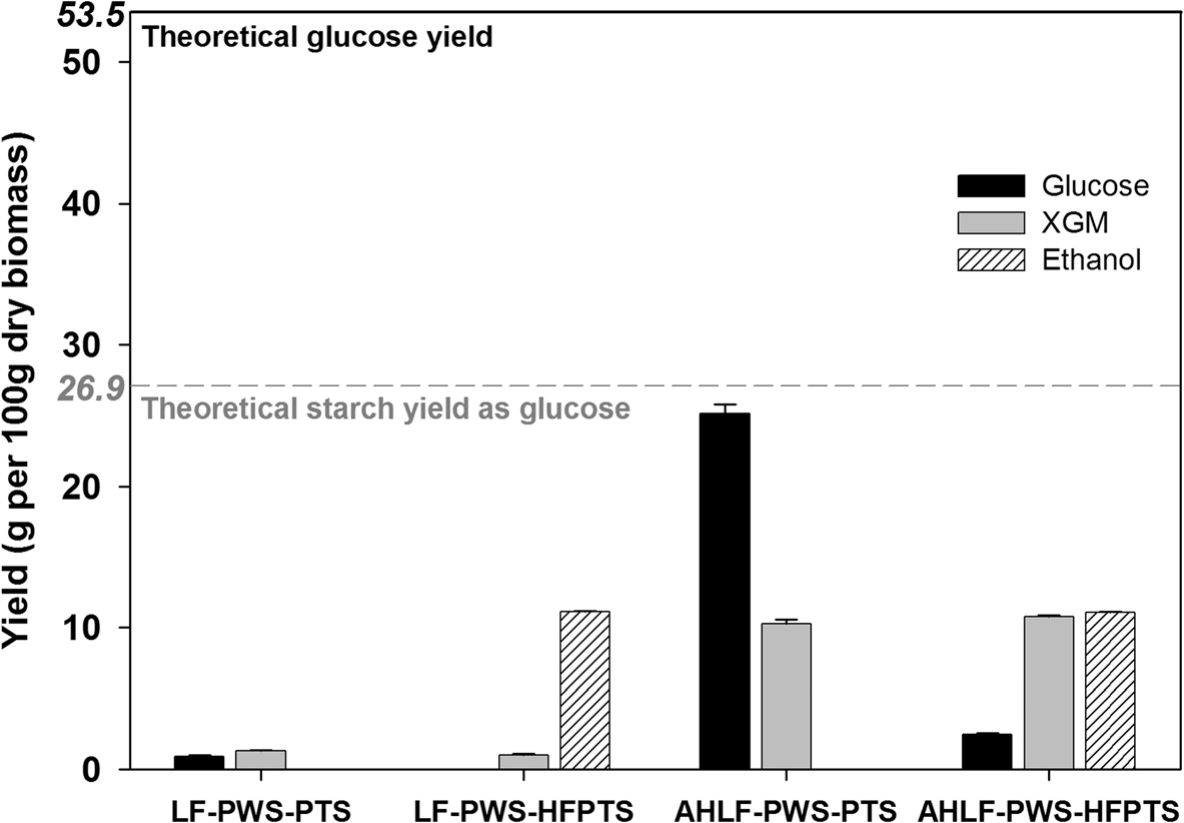
Composition of liquid fractions (LFs) of pretreated whole slurry from autoclaved palm trunk slurry (PWS-PTS) and pretreated whole slurry from pre-hydrolyzed and fermented palm trunk slurry (PWS-HFPTS) before and after 4 % sulfuric-acid-catalyzed hydrolysis of the liquids

The LFs of PWS-PTS and PWS-HFPTS were analyzed by HPLC before and after 4 % sulfuric-acid-catalyzed hydrolysis at 121 °C for 60 min, and the results are presented in Fig. 4. Before acid hydrolysis, there were few monomeric sugars such as glucose and XGM detectable by the HPLC in both the LF-PWS-PTS and LF-PWS-HFPTS. The 11.1 g ethanol was determined in LF-PWS-HFPTS due to pre-conversion of starch to ethanol prior to hydrothermal treatment. After acid hydrolysis, however, glucose and XGM for AHLF-PWS-PTS reached 25.2 and 10.3 g, respectively, which equated to 93.7 % of the theoretical starch yield as glucose and 66.9 % of TXY, respectively. On the other hand, a small amount of glucose was determined in the AHLF-PWS-HFPTS due to pre-conversion of starch to ethanol. However, the 10.9 g of XGM was detected similar to that of AHLF-PWS-PTS. Almost all of the solubilized starch remained as oligosaccharides rather than being converted to glucose under mild acidic condition induced from hydrothermal treatment at 180 °C for 30 min. As described above, oligosaccharides are strong feedback inhibitors that render cellulolytic enzymes less inactive and thereby decelerate the enzymatic digestibility of cellulose. The results regarding their inhibitory effect are discussed further in detail below.

### Effect of cellobiase as a supplement on glucose yield

**Fig. 5:**
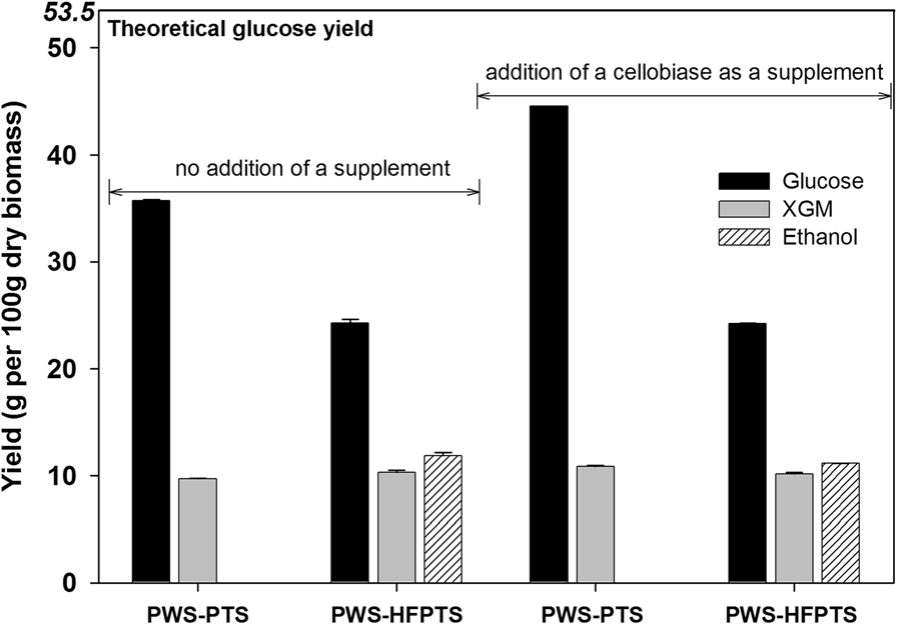
Product yields from pretreated whole slurry from autoclaved palm trunk slurry (PWS-PTS) and pretreated whole slurry from pre-hydrolyzed and fermented palm trunk slurry (PWS-HFPTS) through enzymatic hydrolysis with and without the addition of cellobiase, Novozyme-188, as a supplement

Figure 5 shows the effect on the sugar yields of adding cellobiase as a supplement to enzymatic hydrolysis of PWS-PTS and PWS-HFPTS. Because both PWS-PTS and PWS-HFPTS contain a considerable amount of both hemicellulose and cellulose, enzyme mixtures with a combination of Cellic® CTec2 and Cellic® HTec2 (9 + 1, *v*/*v*) were used for enzymatic hydrolysis. The glucose yield from PWS-PTS through enzymatic hydrolysis with enzyme mixtures of Cellic® CTec2 and Cellic® HTec2 was 35.7 g, which was only 66.7 % of TGY. When a certain amount of cellobiase with activity of 554 CBU/g was added to the enzyme mixtures, however, the resulting glucose yield increased to 44.5 g amounting to 83.2 % of TGY, which was increased by around 25 % compared to that without cellobiase addition. Regardless of the addition of cellobiase, on the other hand, glucose yields from PWS-HFPTSs were 24.3 g, which showed a high rate of enzymatic digestibility of cellulose amounting to 91.4 % of TGY for cellulose. These results imply that the oligosaccharides liberated from starch and hemicellulose in PTS could be inhibitory for Cellic® CTec2 during enzymatic hydrolysis. Adding a small amount of cellobiase induced a synergy effect on enzymatic hydrolysis of PWS-PTS with the enzyme mixtures; thus, enhancing glucose yield could be achieved. In addition, it is expected that there are more possibilities for enhancing glucose yield from OPT and overcoming the feedback inhibition, so more research could be needed to find effective enzyme combination. Consequently, converting starch to ethanol prior to hydrothermal treatment facilitates the conversion of as much cellulose as possible to glucose, which is one of alternatives for maximizing ethanol production from OPT by preventing starch from further degradation during the pretreatment.

### Evaluation of ethanol productivity by CECP and EECP

The product yield profiles by CECP and EECP are summarized in Table 3, respectively. Both processes do not characteristically include washing and solid–liquid separation of pretreated slurry. In EECP, starch was converted to ethanol prior to the addition of CECP. Although CECP also showed favorable sugar and ethanol yield because the mild hydrothermal treatment of OPT restricted further degradation of starch, the enzymatic hydrolysis efficiency of cellulose was more favorable in EECP than that in CECP. The ethanol yield from EECP increased to 25.6 g (93.5 % of theoretical ethanol yield (TEY)), which was higher than the 22.3 g yield of CECP (corresponding to 81.4 % of TEY). On the other hand, the yields of other products were not affected by the applied processes, except for glucose and ethanol yield. In other cases where OPT was biochemically converted to ethanol, when OPT was applied to aqueous alkali pretreatment with 7 % (*w*/*w*) aqueous ammonia for 8 h, followed by simultaneous enzymatic hydrolysis and fermentation of washed pretreated solid, the final ethanol yield was 10.8 g per 100 g of dry biomass, which corresponds to less than 50 % of TEY [21]. The low-ethanol yield could be attributed to removal of the water-soluble fraction containing a considerable amount of starch during washing of the pretreated slurry. Alternatively, another study has focused on maximizing the utilization of starch in OPT. According to Prawitwong et al., OPT was separated into starch-rich PA and VB, after which the PA was autoclaved and then the soluble starch was directly fermented to ethanol [8]. Starch-free PA and VB were subjected to alkali pretreatment with 5 % sodium hydroxide solution at 150 °C for 3 h, followed by enzymatic hydrolysis and fermentation of washed pretreated solid.

**Table 3:** Product yield profiles based on 100 g dry OPT by the conventional ethanol conversion process (CECP) and the efficient ethanol conversion process (EECP)

	Before hydrothermal treatment				After hydrothermal treatment			
Process	Starch hydrolysis		Fermentation		Saccharification		Fermentation	
CECP	Not applicable				Glc	43.5 ± 0.1	Glc	n.d.
					XGM	11.2 ± 0.3	XGM	9.7 ± 0.1
					Ara	1.1 ± 0.0	Ara	0.8 ± 0.0
							EtOH	22.3 ± 0.0
					HMF	0.4 ± 0.0	HMF	0.1 ± 0.0
					FF	0.7 ± 0.0	FF	n.d.
EECP	Glc	25.9 ± 0.1	Glc	n.d.	Glc	25.6 ± 0.1	Glc	n.d.
					XGM	9.6 ± 0.0	XGM	8.7 ± 0.0
					Ara	0.8 ± 0.0	Ara	0.8 ± 0.0
					Acetate	3.5 ± 0.1	Acetate	3.3 ± 0.0
			EtOH	11.9 ± 0.0	EtOH	11.9 ± 0.1^a^	EtOH	25.6 ± 0.1^b^
					HMF	0.2 ± 0.0	HMF	0.1 ± 0.0
					FF	0.9 ± 0.0	FF	n.d.

*n.d.* not detected

^a^Pre-converted ethanol yield during starch fermentation

^b^Final ethanol yield from OPT including pre-converted ethanol

A total of 100 g dry squeezed OPT was able to be separated into 55 g of PA and 45 g of VB. From the designed process, finally, 11.2 g of ethanol was produced from starch in the PA, which accounted for 85.5 % of TEY from starch. From alkali-pretreated starch-free PA and VB, 5.1 and 8.6 g of ethanol were also obtained, respectively. As a result, a total of 24.9 g of ethanol was generated from the squeezed OPT containing a total available glucan content of 59.8 g, which corresponded to 81.6 % of TEY. This ethanol production strategy using OPT designed by Prawitwong et al. has an advantage of minimizing starch loss but could require an expensive process such as biomass fractionation, chemical use/recovery, and washing/conditioning prior to enzymatic hydrolysis and fermentation [8].

Consequently, EECP could be considered as a suitable process for maximizing ethanol production from lignocellulosic biomass such as OPT that has a lot of starch in the biomass. In addition, EECP has an advantage of reducing operational costs due to the simple ethanol production process that excludes washing/conditioning and solid–liquid separation of pretreated slurry prior to enzymatic hydrolysis. The ethanol fermentation by microorganisms capable of digesting both xylose and glucose could further increase the ethanol yield from EECP. Consequently, EECP would become a more effective strategy for conversion of OPT into biofuels and biochemicals.

## Conclusions

The available glucose from 100 g dried OPT reached 53.5 g, comprised of 26.9 g of starch as glucose and 26.6 g of cellulose as glucose. Two different processes were applied to the production of ethanol from OPT. The glucose yield from CECP was 43.5 g (81.3 % of TGY), and the resulting ethanol yield was 22.3 g (81.4 % of TEY). In contrast, EECP converted as much starch and cellulose as possible into glucose, i.e., 25.9 g from the starch and 25.6 g from cellulose, which equated to 96.3 % of TGY. The resulting ethanol yield from EECP of 25.6 g (93.5 % of TEY) was 10 % more than that from CECP. These study results confirmed the potential of EECP for use in maximizing ethanol production from OPT through effective starch utilization while minimizing its loss.

## Abbreviations

AHLF: acid-hydrolyzed liquid fraction
AHLF-PWS-HFPTS: acid-hydrolyzed liquid fraction of pretreated whole slurry from pre-hydrolyzed and fermented palm trunk slurry
AHLF-PWS-PTS: acid-hydrolyzed liquid fraction of pretreated whole slurry from autoclaved palm trunk slurry
Auto-PTS: autoclaved palm trunk slurry
CECP: conventional ethanol conversion process
EECP: efficient ethanol conversion process
HMF: 5-hydroxymethyl-2-furaldehyde
HPLC: high-performance liquid chromatography
HWE: hot-water extractives
LF-PWS-HFPTS: liquid fraction of pretreated whole slurry from pre-hydrolyzed and fermented palm trunk slurry
LF-PWS-PTS: liquid fraction of pretreated whole slurry from autoclaved palm trunk slurry
NREL: National Renewable Energy Laboratory
OPT: oil palm trunk
PA: parenchyma
Pre-HFPTS: pre-hydrolyzed and fermented palm trunk slurry
PWS: pretreated whole slurry
PWS-HFPTS: pretreated whole slurry from pre-hydrolyzed and fermented palm trunk slurry
PWS-PTS: pretreated whole slurry from autoclaved palm trunk slurry
*S. cerevisiae*: *Saccharomyces cerevisiae*
SF: solid fraction
SF-PWS-5 % EtOH: solid fraction of pretreated whole slurry of starch-free palm trunk treated with 5 % ethanol solution at 180 °C for 30 min
SF-PWS-10 % EtOH: solid fraction of pretreated whole slurry of starch-free palm trunk treated with 10 % ethanol solution at 180 °C for 30 min
SF-PWS-HFPTS: solid fraction of pretreated whole slurry from pre-hydrolyzed and fermented palm trunk slurry
SF-PWS-PTS: solid fraction of pretreated whole slurry from autoclaved palm trunk slurry
TEY: theoretical ethanol yield
TGY: theoretical glucose yield
TXY: theoretical XGM yield
VB: vascular bundle
XGM: xylose + galactose + mannose
